# P-409. Use of Point of Care Pediatric Antimicrobial Stewardship Resource and Clinician Perceptions

**DOI:** 10.1093/ofid/ofaf695.626

**Published:** 2026-01-11

**Authors:** Jennifer E Girotto, Ilana Waynik

**Affiliations:** University of Connecticut, Storrs, CT; Connecticut Children's / University of Connecticut, Department of Pediatrics, Hartford, Connecticut

## Abstract

**Background:**

The objective of this project was to assess the impact of Firstline, a unique point of care app (FLPOC) for local antibiogram-based antimicrobial prescribing recommendations, on inpatient and outpatient providers.

**Methods:**

This project was reviewed by the IRB and deemed a quality improvement project. FLPOC was rolled out in stages between month, year and month, year. A survey was developed and emailed to providers at a 205 bed freestanding children’s hospital and to outpatient satellite and referral network providers. Respondents included physicians, advanced practice providers, pharmacists, nurses, and trainees. The survey was sent out in June 2021 prior to FLPOC implementation (BL) and again 40 months after implementation (POST) in November 2024. The survey asked for provider demographics and gaged ease of access of pediatric infectious disease prescribing information [i.e., infections for which the institution has clinical pathways (PATH), inpatient infections without clinical pathways (NP-I), outpatient infections without clinical pathways (NP-O)]. In POST period, providers were additionally asked their frequency of use of the FLPOC app and for recommendations on how to improve the app.

**Results:**

219 and 141 providers responded to the BL and POST surveys respectively. Attending physicians were the most frequent respondents. The top 3 resources used by providers BL and POST implementation included the institution’s clinical pathways (47% BL and 70% POST), Lexicomp (53% BL and 66% POST), FLPOC (51% POST) versus Uptodate (59% BL). For ease of access to antimicrobial information, those that reported extremely easy or very easy improved across all areas (PATH: 44% BL vs 70% POST, NP-I: 15% BL vs 31% POST, NP-O: 26% BL vs 39% POST). The majority of providers used FLPOC at least once or twice monthly. Qualitative data in a comments section was overwhelmingly positive. Recommendations included suggestions to increase provider awareness of the product.

**Conclusion:**

Implementation of FLPOC was highly successful in improving easy access to local antimicrobial stewardship prescribing recommendations. Feedback from providers suggests need for additional education to ensure all providers are familiar with the product.

**Disclosures:**

Jennifer E. Girotto, PharmD, BCPPS, BCIDP, Wolters Kluwer: Advisor/Consultant

